# The Role of Continuous Theta Burst TMS in the Neurorehabilitation of Subacute Stroke Patients: A Placebo-Controlled Study

**DOI:** 10.3389/fneur.2021.749798

**Published:** 2021-11-04

**Authors:** Ana Dionísio, Rita Gouveia, João Castelhano, Isabel Catarina Duarte, Gustavo C. Santo, João Sargento-Freitas, Felix Duecker, Miguel Castelo-Branco

**Affiliations:** ^1^Institute of Nuclear Sciences Applied to Health ICNAS, Coimbra Institute for Biomedical Imaging and Translational Research CIBIT, University of Coimbra, Coimbra, Portugal; ^2^Faculty of Sciences and Technology FCTUC, Department of Physics, University of Coimbra, Coimbra, Portugal; ^3^Faculty of Medicine FMUC, University of Coimbra, Coimbra, Portugal; ^4^Stroke Unit, Neurology Department, Coimbra Hospital and University Centre, Coimbra, Portugal; ^5^Department of Cognitive Neuroscience, Faculty of Psychology and Neuroscience, Maastricht University, Maastricht, Netherlands; ^6^Maastricht Brain Imaging Center, Maastricht University, Maastricht, Netherlands; ^7^Brain Imaging Network, University of Coimbra, Coimbra, Portugal

**Keywords:** continuous theta burst stimulation, transcranial magnetic stimulation, neurophysiology, brain oscillations, stroke, neurorehabilitation

## Abstract

**Objectives:** Transcranial magnetic stimulation, in particular continuous theta burst (cTBS), has been proposed for stroke rehabilitation, based on the concept that inhibition of the healthy hemisphere helps promote the recovery of the lesioned one. We aimed to study its effects on cortical excitability, oscillatory patterns, and motor function, the main aim being to identify potentially beneficial neurophysiological effects.

**Materials and Methods:** We applied randomized real or placebo stimulation over the unaffected primary motor cortex of 10 subacute (7 ± 3 days) post-stroke patients. Neurophysiological measurements were performed using electroencephalography and electromyography. Motor function was assessed with the Wolf Motor Function Test. We performed a repeated measure study with the recordings taken pre-, post-cTBS, and at 3 months' follow-up.

**Results:** We investigated changes in motor rhythms during arm elevation and thumb opposition tasks and found significant changes in *beta* power of the affected thumb's opposition, specifically after real cTBS. Our results are consistent with an excitatory response (increase in event-related desynchronization) in the sensorimotor cortical areas of the affected hemisphere, after stimulation. Neither peak-to-peak amplitude of motor-evoked potentials nor motor performance were significantly altered.

**Conclusions:** Consistently with the theoretical prediction, this contralateral inhibitory stimulation paradigm changes neurophysiology, leading to a significant excitatory impact on the cortical oscillatory patterns of the contralateral hemisphere. These proof-of-concept results provide evidence for the potential role of continuous TBS in the neurorehabilitation of post-stroke patients. We suggest that these changes in ERS/ERD patterns should be further explored in future phase IIb/phase III clinical trials, in larger samples of poststroke patients.

## Introduction

Stroke is the third most frequent cause of death (1) and one of the most prevalent causes of disability (2–4). Motor deficits occur quite often in stroke and affect up to 75% of patients for several months (3, 5–7). In spite of the available interventions, search for alternative therapeutic solutions is an active research area (8, 9).

Transcranial magnetic stimulation (TMS) is under investigation for this purpose, as a potential alternative for the study, diagnosis, and treatment of various diseases given its non-invasive nature with rare adverse effects (10, 11). When applied in its repetitive form, it can produce effects that last beyond the stimulation period (11, 12). Given these effects it might act as a neuromodulatory tool, providing a potential device to restore the balance of activity between the hemispheres, through the modulation of plasticity. In fact, following stroke, it has been postulated that the lesioned hemisphere decreases its activity while the excitability of the unaffected hemisphere becomes pathologically increased (2, 13, 14). Hence, repetitive TMS can be applied to augment the excitability of the stroke-affected hemisphere or to reduce activity in the unaffected hemisphere, depending on stimulation parameters (1, 2, 4, 13).

Although this technique is becoming popular, several issues remain to be elucidated. These include response variability and the still unknown mechanisms behind its application (12, 15). One of the inhibitory protocols that are currently being studied is continuous theta burst stimulation (cTBS), a recent form of patterned TMS that consists of 3 pulses at 50 Hz repeated every 200 ms during 40 sec, inducing inhibitory effects that last up to 60 min (16, 17).

In our previous work in healthy individuals (18), we observed that cTBS induced an unexpected inhibition in the contralateral hemisphere during arm elevation, contradicting the ipsilateral inhibition vs. contralateral disinhibition theory. We hypothesized that this unexpected effect was a result of propagation of effects from the stimulation site, which might have implications for neurorehabilitation. However, it is still possible that such effects only occur in the presence of two healthy hemispheres, and that the theory still holds when one hemisphere is lesioned.

Here we aimed to study the impact of cTBS when applied to the unaffected hemisphere of stroke patients. Cortical activity was evaluated at rest to study the baseline physiological state and during motor tasks, in which concerns brain oscillatory patterns. When sensory information or motor output are absent, there is an inhibition of cortical activity that is observed as an increase in oscillatory activity (event-related synchronization, ERS). In opposition, motor readiness induces an activation observed as a decrease in brain rhythms, designated by event-related desynchronization (ERD) in the *mu* and *beta* bands (19–23). To accomplish our goals, we recorded brain activity using electroencephalography (EEG) to analyze *alpha, mu*, and *beta* rhythms, before (T0) and after (T1) one session of real (experimental group: group E) or sham (control group: group C) cTBS and at 3-months' follow-up (T2, although at this time point we did not expect a change). Moreover, we evaluated motor-evoked potentials, using electromyography (EMG), and motor function, with the Wolf Motor Function Test (WMFT), at the same time points.

## Materials and Methods

We conducted this work in accordance with the Declaration of Helsinki. It has the approval of the Ethics Committee of the Faculty of Medicine of the University of Coimbra. All volunteers gave their written informed consent after explanation of the study procedures and objectives.

### Study Design

This was a proof-of-concept study, wherein only a single-session of cTBS was applied. Patients were randomized in a 1:1 ratio into an active intervention or a placebo group. Subjects allocated to the experimental group (group E) received real continuous theta burst stimulation, while patients who were included in the control group (group C) underwent sham stimulation. Patients, but not investigators, were blinded to group allocation.

### Sample

Patients included in this study were recruited from the Neurology Department of the Coimbra University Hospital and met the following criteria: (1) age between 18 and 85 years, (2) first-ever middle cerebral artery ischemic stroke, (3) cortico-subcortical lesion, (4) time since stroke onset within 7 ± 3 days (subacute phase), (5) upper-limb motor deficit, (6) modified Rankin Scale previous to the stroke event ≤1, and (7) capability to understand the tasks. We excluded subjects that (1) were clinically unstable; had (2) cognitive impairment, (3) diagnosed dementia, (4) history of epilepsy, (5) posterior or global aphasia, (6) neglect; (7) were pregnant, or presented (8) drugs or alcohol abuse, or (9) contraindications to transcranial magnetic stimulation.

Ten right-handed stroke patients that fulfilled the eligibility criteria composed the sample. Clinical and demographic data from the participants are detailed in Table 1.

**Table 1:** Clinical and demographic data of volunteers^a^.

	**Total of participants *N* = 10**	**Group E *N* = 5**	**Group C *N* = 5**
Age (years; mean ± SD)	67.10 ± 13.470	70.20 ± 8.701	64.00 ± 17.564
Gender (female/male)	4 / 6	1/4	3/2
Handedness (points^b^; mean ± SD)	36.00 ± 0.000	36.00 ± 0.000	36.00 ± 0.000
Time since stroke (days; mean ± SD)	8.50 ± 1.581	8.20 ± 1.643	8.80 ± 1.643
Lesion side (right/left hemisphere)	4 / 6	3/2	1/4
NIHSS (mean ± SD)	6.40 ± 3.718	5.60 ± 2.302	7.20 ± 4.919
Baseline WMFT log time (mean ± SD)	2.14 ± 0.651	2.25 ± 0.729	2.04 ± 0.627
Baseline WMFT FAS (points; mean ± SD)	48.80 ± 31.255	45.80 ± 36.341	51.80 ± 29.235

a *FAS, functional ability scale; NIHSS, National Institutes of Health Stroke Scale; WMFT, wolf motor function test; Group E, experimental group; Group C, control group*.

b *All patients scored the maximum (36 points) in an adapted Edinburgh Handedness Inventory questionnaire, which corresponded to being strongly right-handed*.

All the patients who were admitted in this study underwent a prior stroke evaluation protocol at the University Hospital, which included the compilation of demographic information and clinical history, the assessment of stroke severity with the National Institutes of Health Stroke Scale, performed by a neurologist, and neuroimaging investigation reviewed by a neuroradiologist.

### Magnetic Resonance Imaging (MRI)

We started by conducting formal neuroradiological evaluation with structural magnetic resonance imaging to confirm lesion location and characteristics. Data scans were collected on a 3.0 Tesla scanner (Magnetom TIM Trio, Siemens, Erlangen, Germany), equipped with a phased array 12-channel birdcage head coil (Siemens), at the Portuguese Brain Imaging Network Facilities, in Coimbra. We acquired a 3D anatomical T1-weighted MPRAGE (magnetization-prepared rapid acquisition gradient echo) pulse sequence for each patient [repetition time (TR) = 2,530 ms, echo time (TE) = 3.42 ms, inversion time (TI) = 1,100 ms, flip angle (FA) 7°, 176 single-shot slices, voxel size 1 × 1 × 1 mm^3^, field of view (FOV) 256 × 256 mm^2^].

### Wolf Motor Function Test (WMFT)

The motor function of the affected upper-limb was evaluated before (T0), after stimulation (T1), and at 3-months' follow-up (T2), with the WMFT (24). The WMFT consists of an instrument for the assessment of the upper extremity function, in stroke patients. This test combines a series of motor tasks [detailed in (24)], from simple to more complex movements, comprising not only joint-specific but also total limb movements. Speed and quality of the movement are both quantified to evaluate the performance of the upper limb (24). In this work, each patient performed 15 tasks with the affected upper extremity and the performance times were recorded in seconds, with a maximum of 120 s for each task; when the patient could not perform the movement, the time was recorded as 120 s. In addition, we assessed the quality-of-the-movement with the functional ability scale (FAS), where the subject was rated a “0” when the movement was not performed and a “5” when the movement appeared to be normal, with a maximum total of 75 points.

### Electroencephalography (EEG)

The EEG methodology was similar to the one adopted in our previous work in healthy volunteers (18). Briefly, we set up a block-design task, with three conditions performed in the following order: eyes opening/closure, arm movements, and thumb movements. The first condition was composed by 9 blocks × 10 s of eyes opening and 9 blocks × 10 s of eyes closure. For the arm movements, we had 18 blocks of 15 s of activity and another 18 blocks of 15 s without motor activity. The same design was used for the thumb opposition task. The outcomes of the EEG were as follows: *alpha* power for the eyes opening/closure condition; *mu* and *beta* ERD for the movement conditions (arm elevation and thumb opposition tasks). We first recorded cerebral activity at rest, asking the subject to open and close the eyes nine times, keeping the eyes opened/closed for 10 s each block, to evaluate the *alpha* power [8–13 Hz, (25)] as a control outcome measure, in an area far from the stimulation site. Then, we recorded brain activity with motor tasks, namely 90°-arm elevation and thumb opposition. We studied the 10–12 and 15–25 Hz frequency ranges to quantify *mu* and *beta* rhythms, respectively (19, 25–28). Movements were repeated six times with each upper limb and another six with both limbs simultaneously, for 15 s each block and with a no motor activity period between blocks with the same duration. “GO” and “STOP” commands were used to instruct patients to begin and stop the movement, respectively, and online triggers were inserted during the recording. This procedure was implemented at T0, T1, and T2. One of the participants from the control group could not fully perform the EEG protocol, being excluded from the EEG analysis.

The acquisition was performed with a 64-channel EEG cap (QuickCap, Neuroscan, U.S.), using a SynAmps2 RT amplifier and the Scan 4.5 software (Compumedics, Charlotte, NC). We kept impedances below 10 kΩ, added a low-pass filter at 200 Hz and a high-pass filter at DC, and selected a 1,000-Hz sampling rate. After data collection, we performed the following data preprocessing and analysis steps [Scan 4.5 and EEGLAB v.14.1.1b, (29)]. We filtered the signal from 1 to 45 Hz and down-sampled data to 250 Hz. We checked for muscle artifacts and eliminated them. We referenced the data to the average of the channels. After running Independent Component Analysis (ICA), we removed components including blinks and eye movements. For power quantification, custom MATLAB (version R2017b, The MathWorks, U.S.) scripts were implemented [adapted from our previous studies by Castelhano et al. (30) and by Silva et al. (31)], as described in our previous work in healthy participants (18). For quantification purposes, the baseline was defined between −2,000 ms and 0 for the eyes' closure and opening, and between −2,000 and −1,500 ms, before movement, for the motor tasks. *Alpha* power was quantified for the eyes conditions between −2,000 and 10,000 ms. Quantification of motor rhythms (*mu* and *beta* power) was performed between −2,000 ms and 0 ms (pre-movement and preparation) and from 0 to 4,000 ms (time-locked to the start of the early phase of movement execution).

**Figure 1:**
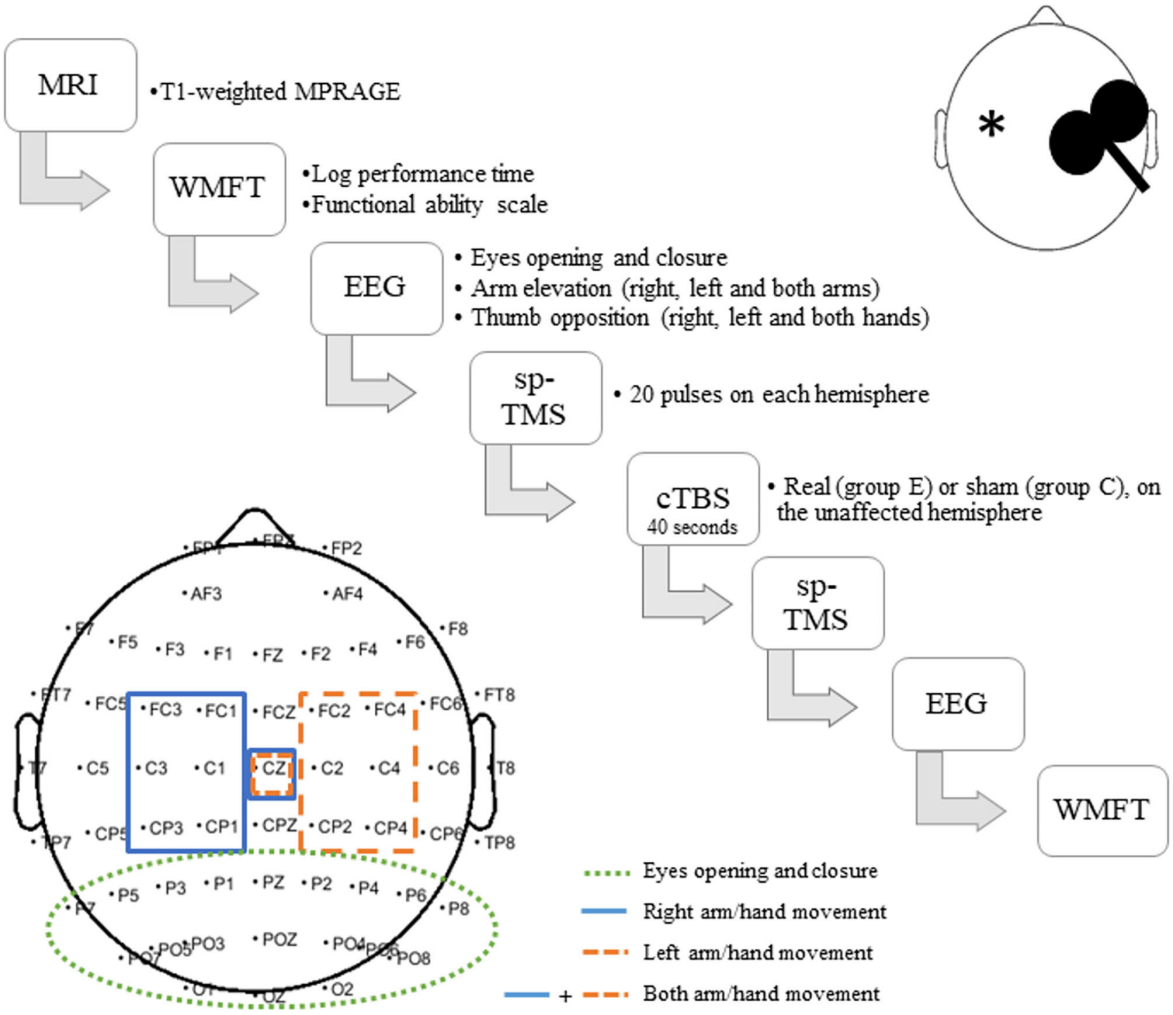
Experimental design and procedures. Group E (Experimental) includes patients who received real stimulation, while in group C (Control) are those patients who received sham stimulation. *Represents the stroke lesion site, which could be either left- or right-sided.

We used posterior electrodes to assess visual *alpha* for the eyes opening/closure condition and central electrodes to study *mu* and *beta* with motor tasks (arm elevation and thumb opposition). The selection of the channels is detailed in Figure 1.

### Electromyography (EMG)

For the recording of electromyographic signal, we first prepared the skin in the areas wherein electrodes would be placed. Then, we placed Ag/AgCl electrodes with conductive paste, in a belly-tendon montage and used BIOPAC MP-150 system and EMG 100C amplifier (Biopac Systems, CA) to record motor-evoked potentials (MEPs) on the abductor pollicis brevis (APB) muscle, using the AcqKnowledge 4.2 software (Biopac Systems, CA) with a 2.500-kHz sampling rate and a 1,000 gain. The peak-to-peak amplitude of motor-evoked potentials was measured *offline* in the same software. Motor-evoked potentials of the unaffected M1 were positive in all participants and we were able to find MEPs of the affected M1 in all patients but three (one from the sham group and two from the real stimulation group).

### Transcranial Magnetic Stimulation (TMS)

Both single-pulse and continuous theta burst were administered with a 70-mm figure-of-eight coil plugged into a MagPro X100 magnetic stimulator (MagVenture, Denmark). All the participants were comfortably seated and wore earplugs during the experiment.

For each hemisphere, we determined the intensity which generated MEPs with a peak-to-peak amplitude ranging from 0.5 to 1 mV and gave 20 single pulses at 100% of the rest intensity determined for the respective hemisphere. Then, we measured MEPs' amplitude, using the same intensity before (T0), 5 min after the cTBS application (T1) and at 3-months' follow-up (T2), to analyze changes in excitability. The cTBS was applied over the motor hotspot of the primary motor cortex of the unaffected hemisphere, at 45° to the sagittal plane, as described in the literature (16, 17), with a total of 600 pulses in 40 s. We defined active motor threshold as being the minimum intensity that triggered at least one minimal muscle twitch on the hand out of three trials, during an isometric contraction, and selected this measure as the intensity for the cTBS protocol (18). We established this number of trials for the active motor threshold definition in order to minimize the discomfort and fatigue associated with the voluntary contraction, since our patients were within the first days after stroke and this task was highly demanding for them. We performed sham stimulation by reducing the intensity to zero level stimulation and using a sham noise generator. All patients from both groups were naïve to TMS and reported perceived real stimulation.

**Figure 2:**
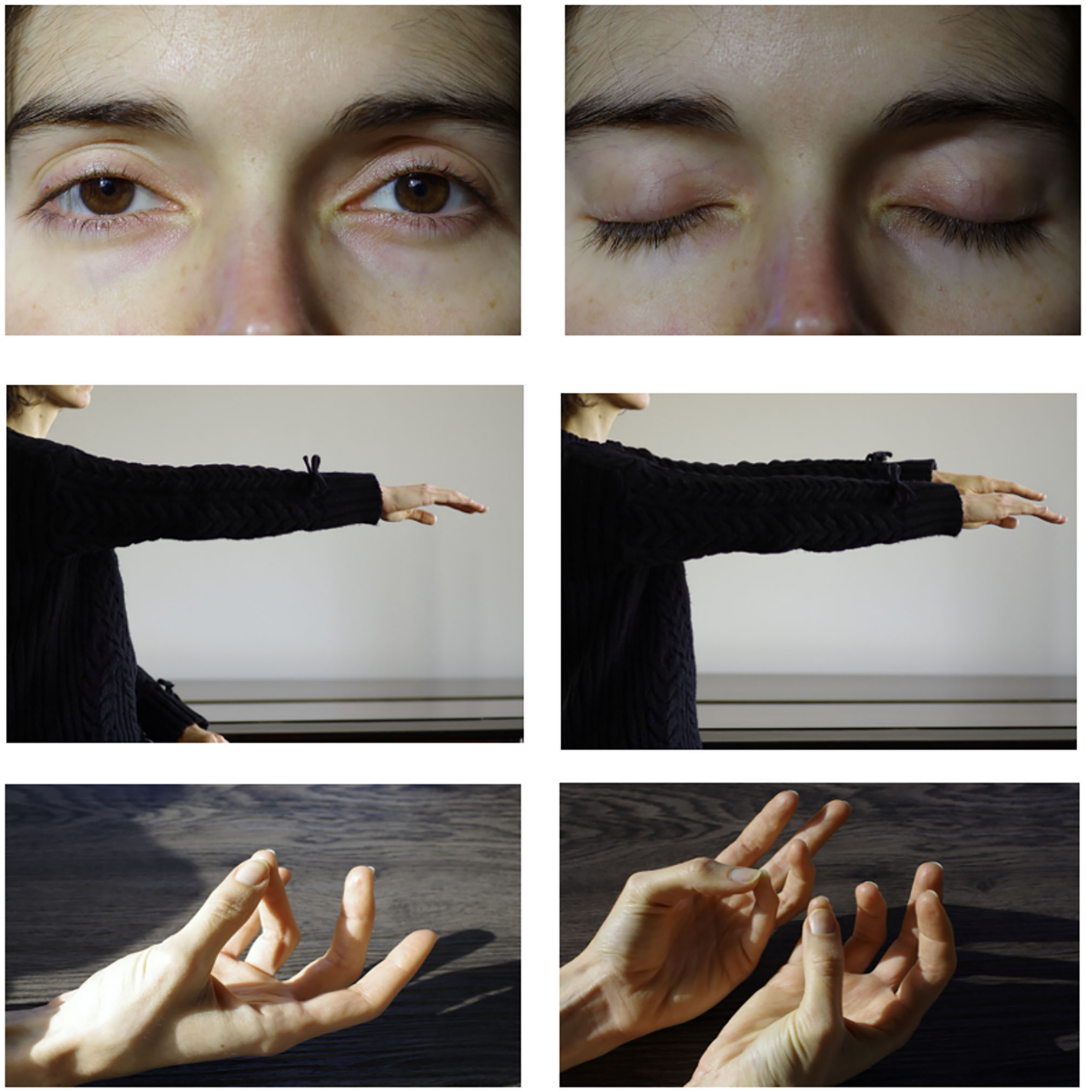
EEG tasks: eyes opening + closure **(top)**, arm elevation **(middle)**, thumb opposition **(bottom)**.

Experimental design is illustrated in Figures 1, 2. All measurements performed after the cTBS, namely EMG, EEG, and WMFT, were acquired within 1 h, which is believed to be the theoretical duration of the neurophysiological effects of cTBS (17).

### Statistical Analysis

Statistical analysis was carried out on SPSS Statistics software v.24 (IBM SPSS Statistics, IBM Corporation, Chicago, IL). For all data, we adopted a 95% confidence interval. Differences between experimental and control groups related to clinical and demographic data were assessed by Mann–Whitney *U*-test, for age, handedness, time-since-stroke onset, National Institutes of Health Stroke Scale at admission, and WMFT baseline measurements, and by the Fisher's exact test, for gender and lesion side. Friedman and Wilcoxon tests were computed to evaluate changes in WMFT, MEPs' amplitude, and mean power of brain rhythms, throughout the three time points (T0, T1, and T2).

## Results

Experimental and control groups were matched. They did not differ significantly regarding age (*U* = 10.500, *p* = 0.730), gender (*p* = 0.524), handedness assessed by Edinburgh Handedness Inventory (32) (*U* = 12.500, *p* = 1.000), lesion side (*p* = 0.524), time-since-stroke (*U* = 10.000, *p* = 1.000), score in the National Institutes of Health Stroke Scale (*U* = 11.500, *p* = 0.881), or WMFT at baseline (log performance time: *U* = 10.000, *p* = 0.690; FAS: *U* = 12.000, *p* = 0.952).

### Magnetic Resonance Imaging

Magnetic resonance structural images were examined by a neuroradiologist, who confirmed the presence of an ischemic unilateral lesion and its location at the vascular territory of the middle cerebral artery.

### Wolf Motor Function Test

WMFT log performance time, which included the duration for all the 15 tasks performed with the affected upper extremity, showed a non-significant reduction trend (Group E: χ^2^ = 4.800, *p* = 0.124; Group C: χ^2^ = 0.500, *p* = 0.931). We observed marginally significant score difference between pre- and post-intervention in Group E (Z = −2.023, *p* = 0.063).

Changes in FAS for the same tasks were not significant [experimental group (E): χ^2^ = 3.125, *p* = 0.259; control group: χ^2^ = 2.286, *p* = 0.370].

**Figure 3:**
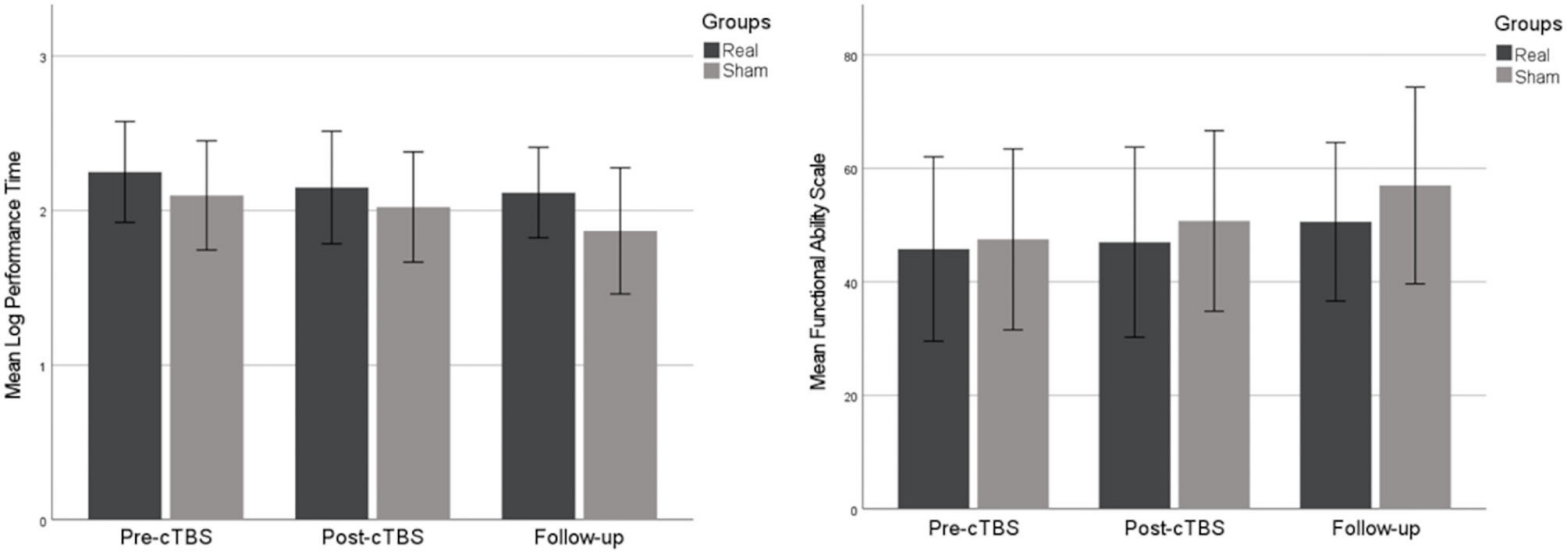
Scores in the Wolf Motor Function Test log performance time **(left chart)** and functional ability scale **(right chart)**, throughout the three time points. Error bars depict ±1 SE.

Results from the WMFT are illustrated in Figure 3.

### Motor-Evoked Potentials

**Figure 4:**
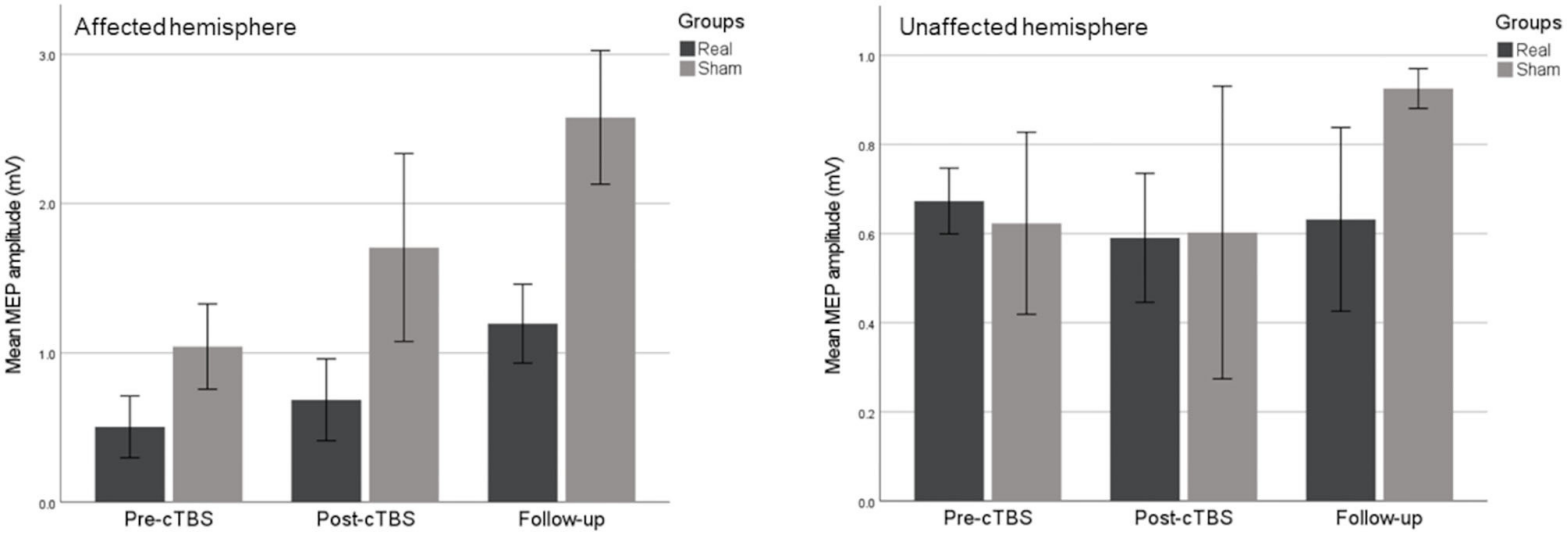
Non-significant changes in motor-evoked potentials of the affected **(left chart)** and unaffected **(right chart)** hemispheres. Error bars show ±1 SE.

Differences were not statistically significant at any time point, concerning MEPs' amplitude of the affected (experimental group: χ^2^ = 4.667, *p* = 0.194; control group: χ^2^ = 4.000, *p* = 0.167) or the unaffected hemisphere (experimental group: χ^2^ = 0.400, *p* = 0.954; control group: χ^2^ = 0.667, *p* = 0.944), as observed in Figure 4.

### Electroencephalography

**Figure 5:**
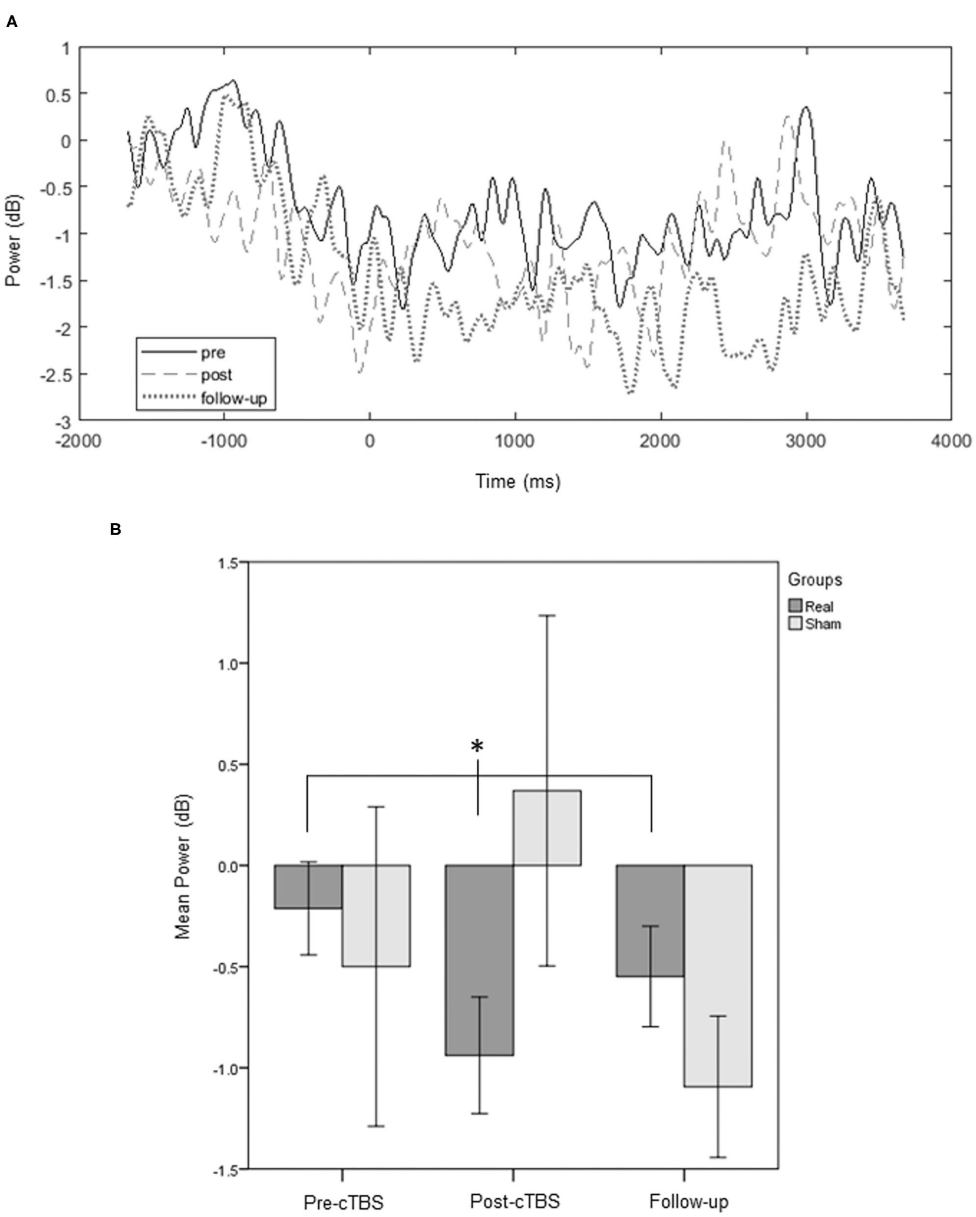
Time-response plots of the mean *beta* power. A group average response of the ipsilesional motor area for an average of the channels of interest (FC3 or 4; FC1 or 2; C3 or 4; C1 or 2; Cz; CP3 or 4; CP1 or 2) is represented, for the experimental group, throughout the three assessment points. Pre-movement and preparation of the affected thumb opposition reveal changes induced by the protocol on *beta* power of the affected hemisphere **(A)**. Significant differences (*p < 0.05) are also illustrated in the bars chart **(B)**. Error bars represent ±1 SE.

Regarding the thumb opposition task, we found a statistically significant change of *beta* rhythm across the three assessment points, in the pre-movement and preparation for movement performed with the affected limb only in the real-stimulation group (Group E: χ^2^ = 6.400, *p* = 0.039, Figure 5; Group C: χ^2^ = 0.667, *p* = 0.944). Wilcoxon test detected, for this group, a trend toward a decrease in *beta* rhythm between T0 and T1, when preparing for the task with the affected limb (Group E: Z = −2.023, *p* = 0.063; Group C: Z = −1.461, *p* = 0.250). For movements of the unaffected thumb or of both thumbs simultaneously we did not detect significant changes (*p* ≥ 0.05).

Concerning bilateral arm elevation, the Wilcoxon test identified a trend toward a significant increase in *beta* power from pre- to post-cTBS in the pre-movement and preparation (Group E: Z = −2.023, *p* = 0.063; Group C: Z = −1.461, *p* = 0.250), and at the early phase of movement execution (Group E: Z = −2.023, *p* = 0.063; Group C: Z = 0.000, *p* = 1.000), only in the experimental group. When assessing movements performed with each arm individually (affected or unaffected), differences were not observed following real or sham stimulation (*p* ≥ 0.05).

Neither visual *alpha* (studied for the eyes condition) nor *mu* rhythm (quantified for the motor tasks) were significantly affected by the stimulation of M1 (*p* ≥ 0.050 in both groups).

## Discussion

This interventional exploratory study is based on the hypothesis that applying an inhibitory TMS protocol to the unaffected hemisphere in stroke will release the lesioned hemisphere from such inhibition. The predicted increase in excitability might potentially help promote recovery (1, 2, 13).

Analyzing our findings, we observed that significant neurophysiological effects were obtained indeed only for the experimental group, post-cTBS, with no measure showing statistical effects for participants who received placebo stimulation. Even marginally significant effects were observed only for the former group.

Regarding motor rhythms, the thumb opposition task revealed significant differences across time measurements for the *beta* band, only for the experimental group, in the pre-movement and preparation for movements performed with the affected hand. A trend toward a significant decrease in *beta* power at T1, in Group E, was suggestive of an excitatory response to the protocol (increase in ERD) (22, 23) from the affected hemisphere, as expected. We also predicted to find changes in the *mu* rhythm, but we did not. Regarding the arm elevation task, we did not detect statistically significant differences following the application of cTBS. We suggest that it is possible that the effect was more pronounced in the thumb task partially because we stimulated the hand representation M1 as a motor hotspot. We also hypothesize that more complex thumb movements potentiate stronger activation of the motor areas (33) in the affected hemisphere, comparing with the unaffected hemisphere or with a healthy brain, leading to better detectability of TMS effects.

Interestingly, motor rhythms did not change significantly during arm elevation or thumb opposition of the unaffected limb alone, after stimulation, which indicates that the protocol can have a larger impact in the hemisphere contralateral to the stimulation thus potentially improving the lesioned hemisphere functional status. This finding was supported by our results in healthy individuals, where we found a significant impact of the cTBS protocol only on the contralateral hemisphere (18).

There is nevertheless an important distinction with the effects observed in healthy participants and subacute stroke patients, concerning the main aim of this study, which was to identify potentially beneficial neurophysiological effects. While in healthy subjects we had observed a significant and paradoxical inhibition of the contralateral hemisphere, for the arm elevation task, in stroke patients we found instead significant excitation expected from the above-mentioned conceptual framework, with thumb opposition. This suggests that changes in cortical excitability in response to distinct neuromodulation protocols may be task-dependent and, more importantly, might be different in health and in disease. We believe that this difference in the effects of cTBS when applied to stroke patients, in comparison with healthy controls, is due to the altered interhemispheric balance following the stroke event, which completely changes underlying physiology [as observed in a previous functional MRI study from our group (34)]. The idea that TMS effects might be influenced by the brain status, particularly the presence of a brain lesion, is highly relevant for neurorehabilitation approaches and warrants future studies to be conducted.

Visual *alpha*, quantified for the posterior electrodes, was not significantly changed by cTBS over M1, as expected.

Electromyographic motor output showed no significant differences in the peak-to-peak amplitude of motor-evoked potentials after stimulation. We consider that this absence of effect might be justified by distinct reasons, as reported and detailed in our previous study in healthy participants (18). Even though the intensity for the application of the cTBS protocol is customarily defined as a function of the active motor threshold, the voluntary contraction could possibly impact the neuromodulation, rendering no effects for the motor-evoked potentials (35, 36). Importantly, the large variability inherent to the measurement of MEPs may have precluded significant changes to be observed. In fact, in opposition to MEPs, EEG oscillations are not predicted to be influenced by remote events such as spinal cord processes, and are thought to produce more consistent responses (37, 38), which might help explain why we have detected statistically significant effects of cTBS with EEG but not with EMG.

We only found trends concerning behavioral data, evaluated in this study by the WMFT of the affected upper extremity, which may be due to the fact that this study mainly aimed at a short-term physiological proof-of-concept in patients with a recent episode of stroke, at a subacute stage. We propose that more stimulation sessions would be needed to obtain significant improvements in the motor function, detectable by the WMFT.

The main limitation of this study is the small sample size, which requires the interpretation of the results to be cautious. The involvement of patients in the first days following the stroke event and the complexity of our study design that was highly demanding in this subacute stage precluded us from including a greater number of patients. Still, our findings provide preliminary evidence on a possible neurophysiological mechanism of action of TMS and, particularly, continuous theta burst stimulation, which might have a great impact in the neurorehabilitation of stroke patients, if supported by future studies conducted in a larger sample of patients.

The neurophysiology of subacute post-stroke patients was changed, consistently with the hypothesis that inhibitory cTBS over the unaffected hemisphere leads to increased excitation of the lesioned hemisphere. Continuous TBS may be useful in stroke neurorehabilitation by altering the ERS/ERD pattern and potentially improving the motor functions, when applied for several sessions. The results from this preliminary work encourage future clinical trials to study the neurophysiological responses to transcranial magnetic stimulation, in particular cTBS, in a specific disease context.

## Data Availability Statement

The raw data supporting the conclusions of this article will be made available by the authors, without undue reservation.

## Ethics Statement

The studies involving human participants were reviewed and approved by Comissão de Ética da Faculdade de Medicina da Universidade de Coimbra. The patients/participants provided their written informed consent to participate in this study. Written informed consent was obtained from the individual(s) for the publication of any potentially identifiable images or data included in this article.

## Author Contributions

AD, RG, GCS, JS-F, FD, and MC-B researched literature and conceived the study. MC-B was involved in gaining ethical approval. MC-B and FD gained funding. AD, RG, GCS, and JS-F were involved in participants' recruitment. AD, RG, and ICD acquired the data. AD, RG, JC, and MC-B made a substantial contribution to data analysis and interpretation. AD wrote the first draft of the manuscript. All authors revised the manuscript critically for important intellectual content and approved the version to be published.

## Funding

This work was supported by Fundação Luso-Americana para o Desenvolvimento [Prémio FLAD Life Sciences 2020] and Portuguese Foundation for Science and Technology (FCT), DSAIPA/DS/0041/2020, FCT-UID /04950/2020, BIGDATIMAGE, CENTRO-01-0145-FEDER-000016—Centro 2020 FEDER, COMPETE, PAC—MEDPERSYST POCI-01-0145- FEDER-016428, and a MSCA—Marie Curie EU grant to FD and MC-B (No. 708492).

## Conflict of Interest

The authors declare that the research was conducted in the absence of any commercial or financial relationships that could be construed as a potential conflict of interest.

## Publisher's Note

All claims expressed in this article are solely those of the authors and do not necessarily represent those of their affiliated organizations, or those of the publisher, the editors and the reviewers. Any product that may be evaluated in this article, or claim that may be made by its manufacturer, is not guaranteed or endorsed by the publisher.
